# Catch-as-catch-can: mRNA vaccination boosts immune responses to SARS-CoV-2 variants

**DOI:** 10.1038/s41392-021-00681-6

**Published:** 2021-07-07

**Authors:** Graham Pawelec, Emilie Picard

**Affiliations:** 1 Department of Immunology, University of Tübingen, Tübingen, Germany; 2 Health Sciences North Research Institute, Sudbury, ON Canada

**Keywords:** Vaccines, Vaccines

In a recent article published in Science, Stamatatos et al.^1^ show that vaccination with mRNA vaccines containing the ancestral form of the SARS-CoV-2 virus boosts cross-variant neutralizing antibodies elicited by infection with that form and to a lesser extent induces such cross-variant antibodies.

A major current global public health concern is whether emerging mutated SARS-CoV-2 variants will escape the excellent degree of protection against disease afforded by prophylactic vaccination either with RNA encoding the viral spike protein or adenoviral vector-mediated vaccines presenting the same target. The recent paper by Stamatatos et al.^1^ goes some way towards answering this question, but the news is both good and bad. They examined 13 SARS-CoV-2-naive individuals twice vaccinated with either the Pfizer/BioNtech BNT162b2 or Moderna mRNA-1273 RNA products, focusing on antibody responses. Both of these RNA vaccines encode a spike protein of the original Wuhan-Hu-1 variant isolated in December 2019 and are expected to elicit similar or identical responses. The important question asked here was whether antibodies raised against this ancestral form would cross-react on B.1.351 variants which originally arose in South Africa and have since rapidly spread, displacing the original lineage in many countries. Responses were assessed by measuring IgG, IgA and IgM antibody titers, and testing the neutralising capacity on Wuhan-Hu-1 and B.1.351 pseudoviruses. These parameters were compared with the behaviour of anti-spike antibodies from 15 individuals who had recovered from confirmed SARS-CoV-2 infection, and had antibodies in serum collected before and after a single mRNA vaccination. Several important observations were made in this study: (1) two doses of mRNA vaccine administered to previously unexposed individuals did result in the production of neutralising antibodies against B.1.351 variants but at far lower titers than against the ancestral Wuhan-Hu-1 variant; (2) only one-third of previously infected individuals possessed neutralising antibodies against B-1-351 whereas 80% had antibodies against Wuhan-Hu-1, the strain with which they had presumably been infected; (3) after vaccination with mRNA vaccines, 87% of previously infected individuals showed increased antibody titers, but again these were lower against B.1.351 than Wuhan-Hu1; (4) previously infected individuals without neutralising antibody did not generate such antibody after vaccination, correlating with a lack of memory B cells specific for spike protein; (5) although the identified cross-neutralising antibodies were directed to the receptor-binding domain, mutation of the N-terminal domain also impacted on the sensitivity of the variants to neutralisation; (6) importantly, but not investigated in detail, spike-specific CD4+ T cells were present in all previously infected donors and were boosted by vaccination; (7) potentially of even greater importance, spike-specific CD4+ T cells were detected at equally high levels in twice-vaccinated uninfected individuals.

These data raise many interesting questions of practical concern, especially whether neutralising antibody titers are correlates of clinical protection as would be expected from findings that equivalent titers against Wuhan-Hu-1 are 95% protective against COVID-19 in phase III trials. The critical question remains whether the lower titers against the B.1.351 variant would also be protective. This cannot be estimated at the moment, but the authors point out that even low titers of neutralising antibodies seem able to protect non-human primates against SARS-CoV-2-challenge, especially when CD8+ T cells are also present.^2^ These findings make it even more important to extend the studies presented by Stamatatos et al. to include not only the CD4+ T cells but also CD8+ T cells and potentially other components of cellular in addition to innate immunity.^3^

Another important question raised by the Stamatatos et al. study is why immune memory of a natural infection appears to be more effective than the response elicited by the mRNA vaccines. This phenomenon has also been observed with seasonal influenza vaccination where vaccinated individuals who develop influenza illness show a more robust response to a subsequent influenza vaccination after recovery than people who did not develop influenza in the prior season. This suggests that actual infection, in contrast to vaccination, can effectively re-stimulate immune memory and implies that there is something missing in the immune response to the vaccine.^4^ It is conceivable that the weaker responses of twice-vaccinated SARS-CoV-2-unexposed individuals might be increased further on booster vaccinations at a later time with the same vaccine, or possibly with modified mRNA sequences, or other vaccine technology, currently in development.

**Fig. 1:**
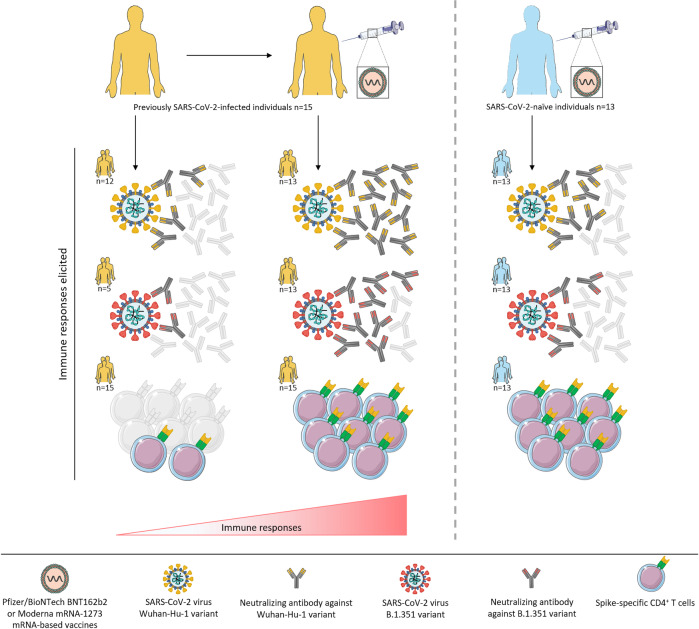
Immune responses to SARS-CoV-2 variants are elicited following mRNA vaccination and are further increased in previously SARS-CoV-2-infected and recovered individuals after vaccination. Neutralizing antibody titers against Wuhan-Hu-1 and B.1.351 along with the frequency of spike-specific CD4+ T cells are greatly enhanced in previously SARS-CoV-2-infected individuals subsequent to mRNA vaccination. Neutralizing antibody titers against both the Wuhan-Hu-1 vaccine antigen and the B.1.351 variant are higher than in SARS-CoV-2-naive individuals who received either Pfizer/BioNTech BNT16b2 or Moderna mRNA-1273 vaccine, while an equally high level of spike-specific CD4+ T cells is detected in both vaccinated groups. Overall, neutralizing antibodies against B.1.351 variants are present but at lower titers than against Wuhan-Hu-1 variants in all groups of individuals regardless of vaccination

Clearly, there are many limitations to the extensive serological analysis by Stamatatos et al.,^1^ most obviously that only a very small number of individuals was tested, and any sex-associated differences could not be taken into account, although it is known that sex plays an important role in clinical outcome. The age range of the subjects was 40–63 years only, and again, age is a major factor of concern in the clinical course of COVID.^5^ Also, the effects of ethnicity could not be investigated. Thus, study participant selection was based on availability and there were no specific inclusion or exclusion criteria. Nonetheless, or perhaps because of these limitations, the study points to SARS-CoV-2 responses likely to be generalisable across populations, and at the same time adds important data to our knowledge of the heterogeneity of immune responses to natural infection and to vaccination that require further exploration. On the other hand, Stamatatos et al. tested only the B1.351 variant, but there are other known and emerging variants. However, the B.1.1.7 (UK) and B1.351 variants share several crucial mutations, particularly in the spike receptor-binding domain and those affecting spike density on the virion, which suggests a least some degree of cross-reactivity on variants other than only B.1.351. This remains to be tested, as does the issue of whether vaccination using platforms other than lipid nanoparticle mRNA vaccines, particularly the chimpanzee adenovirus-vectored vaccine, elicit the same degree of cross-variant response. Nevertheless, the overriding crucial message of this paper remains that whether or not they have been previously infected with the virus, as many people as possible should be vaccinated in an effort to protect them not only against ancestral SARS-CoV-2 but also at least to some extent against emergent variants Fig. 1.
